# Bis-Lactam Peptide [*i*, *i*+4]-Stapling with α-Methylated Thialysines

**DOI:** 10.3390/molecules25194506

**Published:** 2020-10-01

**Authors:** Bo Wu, Weiping Zheng

**Affiliations:** School of Pharmacy, Jiangsu University, 301 Xuefu Road, Zhenjiang 212013, Jiangsu Province, China; 2211615017@stmail.ujs.edu.cn

**Keywords:** peptide stapling, bis-lactam [*i*, *i*+4]-stapling, α-methyl-thialysine, % α-helicity, proteolytic stability

## Abstract

Four bis-lactam [*i*, *i*+4]-stapled peptides with d- or l-α-methyl-thialysines were constructed on a model peptide sequence derived from p110α[E545K] and subjected to circular dichroism (CD) and proteolytic stability assessment, alongside the corresponding bis-lactam [*i*, *i*+4]-stapled peptide with l-thialysine. The % α-helicity values of these four stapled peptides were found to be largely comparable to each other yet greater than that of the stapled peptide with l-thialysine. An l-α-methyl-thialysine-stapled peptide built on a model peptide sequence derived from ribonuclease A (RNase A) was also found to exhibit a greater % α-helicity than its l-thialysine-stapled counterpart. Moreover, a greater proteolytic stability was demonstrated for the l-α-methyl-thialysine-stapled p110α[E545K] and RNase A peptides than that of their respective l-thialysine-stapled counterparts.

## 1. Introduction

Peptide stapling refers to the formation of an intra-chain macrocycle on a parent linear peptide, thereby inducing and stabilizing an α-helical conformation [1,2,3,4,5,6,7,8]. Stapled peptides have mostly been constructed with the [*i*, *i*+4]- or the [*i*, *i*+7]-stapling systems [9], and to a lesser extent with the [*i*, *i*+11]- or the [*i*, *i*+3]-stapling systems [10,11,12,13]. Due to its capability of potentially enhancing the proteolytic stability, cell permeability, and target binding affinity of a parent linear peptide, the peptide stapling technology has found extensive and successful use in chemical biology and medicinal chemistry [1,2,3,4,5,6,7,8]. While all-hydrocarbon stapling with the α-methyl-α-alkenyl α-amino acids including (*S*)-2-(4′-pentenyl)alanine and (*R*)-2-(7′-octenyl)alanine has been the prototypical and most extensively used peptide stapling strategy [9], it is interesting to note that all-hydrocarbon [*i*, *i*+4]-stapling with (*S*)-2-(4′-pentenyl)alanine (an α-disubstituted α-amino acid) on a linear peptide sequence derived from the BH3 domain of the BH3-only BCL-2 family member BID was found not to be more able to stabilize the α-helical conformation of the parent linear peptide than that with the corresponding α-monosubstituted amino acid (i.e., (*S*)-2-(4′-pentenyl)glycine) [14]. In the current study, we were interested in examining the outcome of employing α-methyl-thialysines with bis-lactam peptide [*i*, *i*+4]-stapling and would like to see if the use of α-methyl-thialysine (an α-disubstituted α-amino acid) or thialysine (an α-monosubstituted α-amino acid) in such a stapling system on a peptide sequence might also yield comparable % α-helicity. To this end and as detailed below in Section 2.1., we chose a peptide sequence derived from p110α[E545K] (an oncogenic E545K variant of p110α, the catalytic subunit of phosphatidylinositol 3-kinase α (PI3Kα) [15]) as our model peptide sequence, since this sequence was the one upon which the up-to-date most efficacious bis-lactam [*i*, *i*+4]-stapling mode was developed recently in our laboratory [16].

## 2. Results and Discussion

### 2.1. Stapled Peptides 2–6: Design

**Figure 1:**
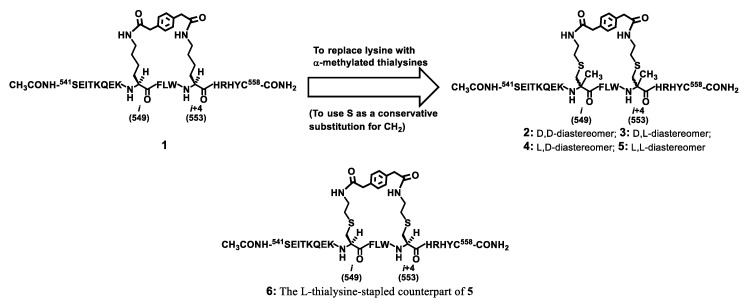
The design of the stapled peptides **2**–**6**, analogs of the stapled peptide **1** that our laboratory discovered recently [16].

Our laboratory recently discovered with a model peptide sequence corresponding to amino acids 541–558 of p110α[E545K] that the bis-lactam [*i*, *i*+4]-stapling with *N*^ε^-*para*-phenylenediacetyl-lysine was able to afford a decent % α-helicity, which represents the up-to-date most efficacious bis-lactam [*i*, *i*+4]-stapling mode [16]. As depicted in Figure 1, starting with such a bis-lactam [*i*, *i*+4]-stapled peptide (i.e., **1**), we would like to assess the outcome of stapling with the α-methylated amino acids at the two stapling sites. For this, while the use of α-methyl-lysine in lieu of lysine would be the most direct replacement, in the current study we prepared the bis-lactam [*i*, *i*+4]-stapled peptides **2**–**5** depicted in Figure 1 that harbored either d- or l-α-methyl-thialysine (conservative replacement for either d- or l-α-methyl-lysine) at a stapling site, for the sake of synthetic accessibility and our desire to replace the two lysine residues in **1** with either d- or l-α-methylated analogs.

It should be noted that the stapled peptide **6** depicted in Figure 1 was also prepared in the current study for a direct comparison of its % α-helicity and proteolytic stability with those of the stapled peptide **5**, since **5** is the analog among **2**–**5** that displays the l-residue at both stapling sites so it was picked for a direct comparison with its l-thialysine-stapled counterpart (i.e., **6**) for their % α-helicity and proteolytic stability.

### 2.2. Stapled Peptides 2–6: Preparation

**Scheme 1:**
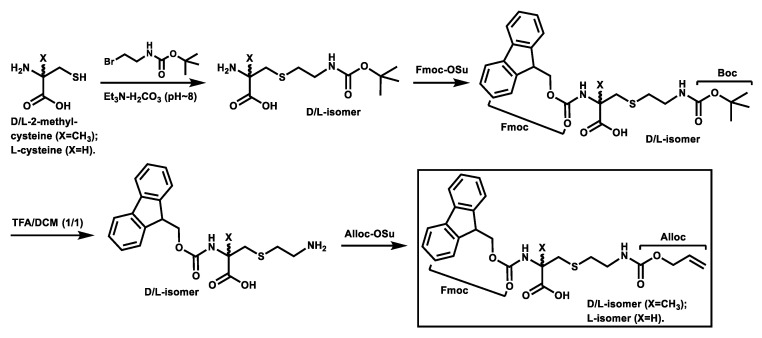
Synthesis of the Fmoc,Alloc-diprotected d-/l-α-methyl-thialysines and the Fmoc,Alloc-diprotected L-thialysine.

**Scheme 2:**
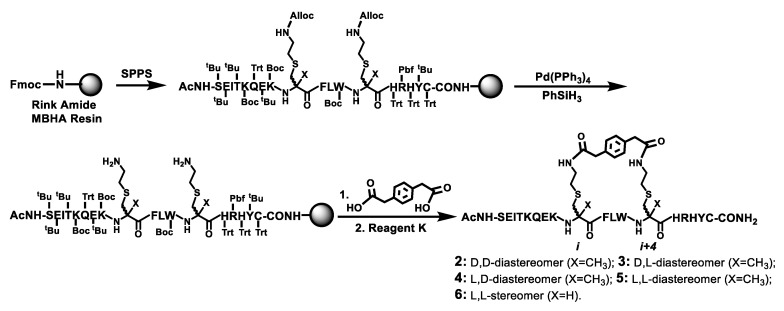
The solid phase synthesis of the stapled peptides **2**–**6** on Rink amide MBHA resin. The on-resin bis-lactamization between the two free amino groups on the resin and *para*-phenylenediacetic acid was performed with HBTU in the presence of 0.4 M NMM/DMF. Ac, acetyl; Reagent K, a TFA-containing peptide cleavage cocktail.

Scheme 1 depicts the synthesis of the Fmoc,Alloc-diprotected d-/l-α-methyl-thialysines and the Fmoc,Alloc-diprotected l-thialysine [17] that were used subsequently for the synthesis of the stapled peptides **2**–**6** based on solid phase peptide synthesis (SPPS) depicted in Scheme 2.

As depicted in Scheme 1, the intermediate from the initial S-alkylation of d-2-methyl-cysteine (or l-2-methyl-cysteine or l-cysteine) with 2-(*tert*-butoxycarbonylamino)ethyl bromide in the presence of an 1.0 M triethylammonium bicarbonate buffer (pH ~8) was treated with *N*-(9-fluorenylmethoxycarbonyloxy)succinimide (Fmoc-OSu) and the resulting Fmoc-protected intermediate was treated with a 50% (*v/v*) solution of trifluoroacetic acid (TFA) in dichloromethane (DCM). The resulting intermediate with a free side chain amino group was then treated with *N*-(allyloxycarbonyloxy)succinimide (Alloc-OSu), affording the Fmoc,Alloc-diprotected d- and l-α-methyl-thialysines and the Fmoc,Alloc-diprotected L-thialysine in overall yields of ~25–31%. It should be noted that the synthesis of the Fmoc,Alloc-diprotected l-thialysine was also reported previously by Meledin, et al. [17], however, our synthetic route seems to be more straightforward than theirs, yet without a dramatic sacrifice of the overall yield.

Scheme 2 depicts the synthesis of the stapled peptides **2**–**6** by the Fmoc chemistry-based manual SPPS on the Rink amide 4-methylbenzhydrylamine (MBHA) resin. Following assembling on the resin the linear peptide with all the side chain protecting groups, the two Alloc groups at *i* and *i*+4 positions were selectively removed with phenylsilane (PhSiH_3_) and tetrakis(triphenylphosphine)palladium (Pd(PPh_3_)_4_) [18]. The two exposed free amino groups were then reacted with *para*-phenylenediacetic acid to realize the on-resin bis-lactamization. The final treatment of the resulting peptidyl-resin with Reagent K afforded the crude stapled peptides **2**–**6**, which were each purified by semi-preparative reversed-phase high performance liquid chromatography (RP–HPLC). Their exact masses were confirmed with mass spectrometry (MS) analysis (Table 1). The purified **2**–**6** were obtained in overall yields of ~1.2–2.4% and each was >95% pure based on a RP–HPLC analysis.

### 2.3. Stapled Peptides 2–6: CD Measurement

**Figure 2:**
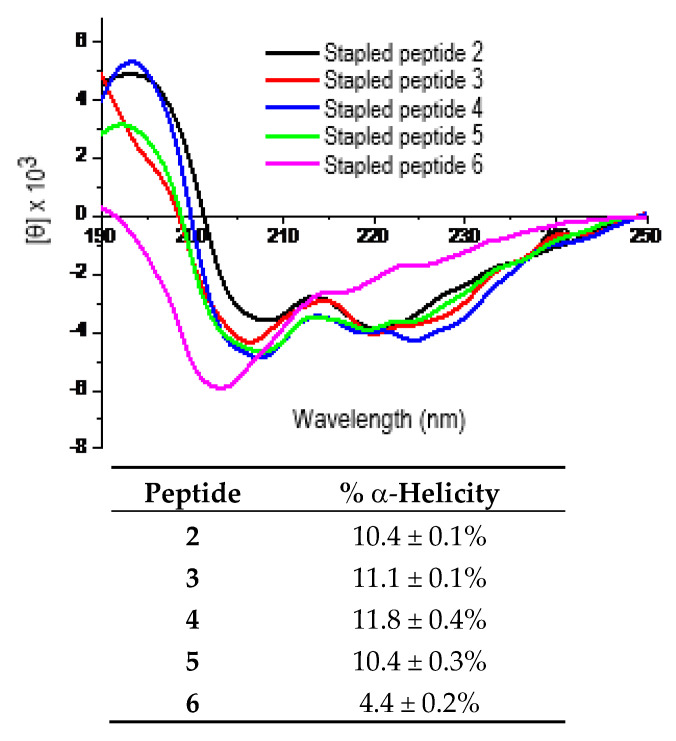
Representative CD spectra of the stapled peptides **2**–**6** (each at 25 μM in ddH_2_O, 25 °C) and the calculated % α-helicity values. Each peptide was assayed in duplicate at 25 µM and/or 50 μM in ddH_2_O. The mean residue ellipticity ([θ]) and the % α-helicity values were calculated according to the literature equations [19]. Note: The % α-helicity values (mean ± standard deviation) were calculated for peptides **2**–**6** from the [θ] values at 222 nm (i.e., [θ]_222_) and the maximum [θ]_222_. See Experimental Section for experimental details.

The purified peptides **2**–**6** were then subjected to circular dichroism (CD) measurement. Each peptide was assayed in duplicate at 25 μM and/or 50 µM in double de-ionized water (ddH_2_O). Figure 2 shows the representative CD spectra for these five peptides each at 25 µM in ddH_2_O and the calculated % α-helicity values. As shown, the stapled peptides **2**–**5** exhibited largely comparable α-helicity values. However, the % α-helicity values of **2**–**5** were ~2.4–2.7-fold greater than that of **6**. These findings indicated that the use of d-α-methyl-thialysine or l-α-methyl-thialysine at *i* and *i*+4 positions of the p110α[E545K] model peptide sequence apparently did not have a significant impact on the propensity of the peptide sequence to adopt an α-helical conformation in water. More importantly, they also indicated that the use of l-α-methyl-thialysine in lieu of l-thialysine with the bis-lactam peptide [*i*, *i*+4]-stapling on the p110α[E545K] model peptide sequence was able to bring about an increase in % α-helicity in water.

### 2.4. Stapled Peptides 5 and 6: Proteolytic Stability Assessment

We further compared the proteolytic stability of the l-α-methyl-thialysine-stapled peptide **5** and the l-thialysine-stapled peptide **6** by employing pronase as the proteolytic enzyme preparation in a proteolysis experiment. Of note, pronase is a mixture of a variety of different proteases/peptidases and therefore has a very broad substrate specificity [20]. We found that **5** was proteolytically more stable than **6**. Specifically, even though **5** and **6** were both completely degraded following 15 min of pronase digestion, ~19.2% and ~5.6% of **5** remained respectively following 4 min and 7.5 min of pronase digestion, whereas the percentages of **6** remained were ~11.1% and ~4.3% respectively following 4 min and 7.5 min of pronase digestion. This observed difference in the proteolytic stability of **5** and **6** is in the same direction of change with the % α-helicity of **5** and **6** (see above), which would be consistent with the notion that an enhanced % α-helicity would improve the proteolytic stability of a stapled peptide, which was also supported previously with the peptides stapled with other stapling systems such as the all-hydrocarbon-stapled peptides [5,9,14].

### 2.5. Stapled Peptides 7 and 8: Preparation, CD Measurement, and Proteolytic Stability Assessment

To preliminarily address if our finding with the stapled peptides **5** and **6** that the use of l-α-methyl-thialysine in lieu of l-thialysine with the bis-lactam peptide [*i*, *i*+4]-stapling was able to afford enhanced % α-helicity and proteolytic stability could be extended to other peptide sequences, we further prepared the l-α-methyl-thialysine-stapled peptide **7** and the l-thialysine-stapled peptide **8** based on another model peptide sequence, i.e., that derived from ribonuclease A (RNase A) also employed previously by Schafmeister, et al. [9].

**Figure 3:**
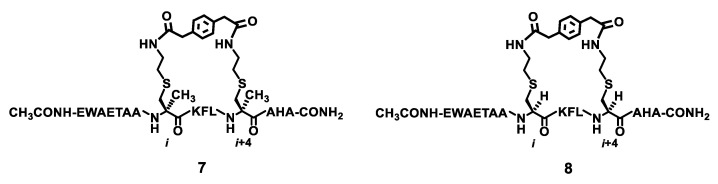
The chemical structures of the stapled peptides **7** and **8**.

The stapled peptides **7** and **8** (depicted in Figure 3) were prepared in the same manner as that for the stapled peptides **2**–**6** described above and in Scheme 2. The prerequisite Fmoc,Alloc-diprotected l-α-methyl-thialysine and the Fmoc,Alloc-diprotected l-thialysine were also prepared according to Scheme 1. The crude peptides **7** and **8** were also purified by RP–HPLC, and their exact masses were also confirmed with MS analysis (Table 1). The purified **7** and **8** were each also >95% pure based on a RP–HPLC analysis. Of note, the purified **7** and **8** were obtained in overall yields of ~1.5% and ~0.12%, respectively.

**Figure 4:**
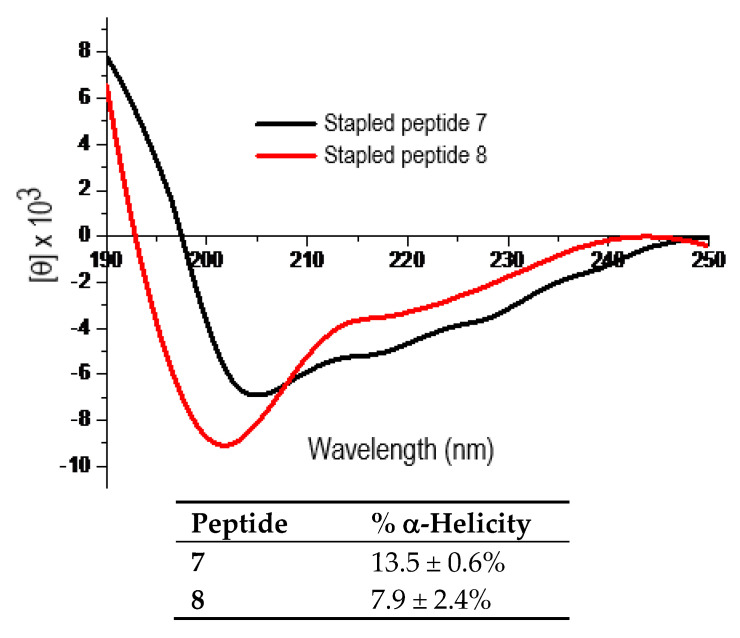
The representative CD spectra of the stapled peptides **7** and **8** (each at 25 µM in ddH_2_O, 25 °C) and the calculated % α-helicity values. Each peptide was assayed in duplicate at 25 µM and/or 50 µM in ddH_2_O. The mean residue ellipticity ([θ]) and the % α-helicity values were calculated in the same manner as that for peptides **2**–**6** according to the literature equations [19]. See Experimental Section for experimental details.

Figure 4 shows the representative CD spectra for the purified peptides **7** and **8** each at 25 µM in ddH_2_O and the calculated % α-helicity values. As shown, the l-α-methyl-thialysine-stapled peptide **7** exhibited ~1.7-fold greater % α-helicity than the l-thialysine-stapled peptide **8**. The subsequent proteolysis experiment with pronase as the proteolytic enzyme preparation revealed that **7** was also proteolytically more stable than **8**. Specifically, while ~96.6%, ~93.3%, and ~81.3% of **7** remained respectively following 4 min, 7.5 min, and 15 min of pronase digestion; ~48.9%, ~35.7%, and ~21.6% of **8** remained respectively following 4 min, 7.5 min, and 15 min of pronase digestion.

## 3. Experimental Section

### 3.1. General

The following materials were obtained from commercial sources for compound synthesis/purification and the pronase digestion assay, and were used as received. Sigma-Aldrich China (Shanghai, China): *N*-methylmorpholine (NMM), trifluoroacetic acid (TFA), *N,N*-dimethylformamide (DMF), *N*-(allyloxycarbonyloxy)succinimide (Alloc-OSu), triethylamine, Trizma, the pronase from *Streptomyces griseus*; TCI Shanghai: *para*-phenylenediacetic acid, 2-(*tert*-butoxycarbonylamino)ethyl bromide, 1,8-diazabicyclo[5.4.0]undec-7-ene (DBU), l-cysteine, *N*-(9-fluorenylmethoxycarbonyloxy) succinimide (Fmoc-OSu), phenylsilane (PhSiH_3_), tetrakis(triphenylphosphine)palladium (Pd(PPh_3_)_4_); Alfa Aesar China (Shanghai, China): 2-(1*H*-benzotriazole-1-yl)-1,1,3,3-tetramethylaminium hexafluorophosphate (HBTU), 2-(7-Aza-1*H*-benzotriazole-1-yl)-1,1,3,3-tetramethyluronium hexafluorophosphate (HATU), *N*-hydroxybenzotriazole (HOBt), phenol, thioanisole, ethanedithiol; Honeywell China (Shanghai, China): acetonitrile, dichloromethane (DCM), methanol, ethyl acetate; Sinopharm Chemical Reagent Co., Ltd. (Shanghai, China): acetic anhydride, diethyl ether, semi-preparative thin-layer chromatography (TLC) silica gel plate (HSGF254, 20 × 20 cm, 0.4–0.5 mm); Shanghai Plus Bio-Sci & Tech Co., Ltd. (Shanghai, China): Rink amide 4-methylbenzhydrylamine (MBHA) resin; SyncoZymes Co., Ltd. (Shanghai, China): d-2-methyl-cysteine hydrochloride; Fisher Scientific Worldwide Co., Ltd. (Shanghai, China): l-2-methyl-cysteine hydrochloride.

The Fmoc,Alloc-diprotected d-/l-α-methyl-thialysines and the Fmoc,Alloc-diprotected l-thialysine were prepared as described below, all the other *N*^α^-Fmoc-protected amino acids for the Fmoc chemistry-based manual solid phase peptide synthesis (SPPS) on the Rink amide MBHA resin were purchased from Sigma–Aldrich China, Alfa Aesar China, TCI Shanghai, or GL Biochem (Shanghai) Ltd.

Unit-resolution mass spectrometry (MS) data were recorded on a Thermo LXQ LC-ion trap mass spectrometer (Thermo Scientific, Waltham, MA, USA) at Jiangsu University. High-resolution mass spectrometry (HRMS) data were recorded on an AB 5600+ Q TOF high-resolution mass spectrometer (AB Sciex LLC, Framingham, MA, USA) at the Pharmacy School of Fudan University. Analytical and semi-preparative reversed-phase high performance liquid chromatography (RP–HPLC) were performed on a Shimadzu LC-20AT station (Shimadzu, Nakagyo-ku, Kyoto, Japan). The circular dichroism (CD) spectra were recorded on a JASCO circular dichroism spectrometer (Model J-815, JASCO Corporation, Tokyo, Japan) at Jiangsu University. The ^1^H- and ^13^C-NMR spectra were recorded on a Bruker Avance II 400 MHz NMR spectrometer (Bruker Corporation, Billerica, MA, USA) at Jiangsu University and the chemical shift values (δ, ppm) were assigned relative to the water peak (δ = 4.87 ppm) in all ^1^H-NMR spectra or relative to the solvent (CD_3_OD) peak (δ = 49.15 ppm) in all ^13^C-NMR spectra.

### 3.2. Compound Preparation

#### 3.2.1. Preparation of the Fmoc,Alloc-diprotected d-/l-α-methyl-thialysines and the Fmoc,Alloc-diprotected l-thialysine 

(a) To a stirred solution of 2-(*tert*-butoxycarbonylamino)ethyl bromide (1 mmol) in methanol (freshly degassed with nitrogen gas) (33 mL) was added dropwise a solution of d-2-methyl-cysteine hydrochloride (or l-2-methyl-cysteine hydrochloride or l-cysteine) (1 mmol) in 1.0 M triethylammonium bicarbonate buffer (pH ~8) (33 mL) at room temperature. After the addition was complete, the reaction mixture was stirred under a nitrogen atmosphere at room temperature for 3 days (or 12 h for the reaction with l-cysteine). The reaction mixture was then concentrated under reduced pressure to remove methanol and extracted with ethyl acetate to remove any possible unreacted 2-(*tert*-butoxycarbonylamino)ethyl bromide. The remaining aqueous solution was lyophilized overnight. (b) To the obtained residue was added ddH_2_O (5 mL) and a 10% (*w*/*v*) aqueous solution of Na_2_CO_3_ (5 mL), and to the resulting solution was then added dropwise while stirring a solution of Fmoc-OSu (2 mmol) in 1,4-dioxane (5 mL) at room temperature. The reaction mixture was subsequently stirred at room temperature overnight, diluted with the addition of ddH_2_O (10 mL), extracted with diethyl ether (3 × 10 mL) to remove excess Fmoc–OSu, acidified at 0 °C with a 5% (*w*/*w*) aqueous solution of tartaric acid, and extracted with ethyl acetate (3 × 10 mL). The combined organics were washed with ddH_2_O and brine, dried over anhydrous Na_2_SO_4_, filtered, and concentrated under reduced pressure. The Fmoc,Boc-diprotected intermediates in the respective residues thus obtained were preliminarily isolated by semi-preparative silica gel TLC developed with methanol/DCM (1/6, *v/v*), and the desired intermediates were each eluted out of the excised silica gel band also with methanol/DCM (1/6, *v*/*v*). The Fmoc,Boc-diprotected d-α-methyl-thialysine: ESI–MS calculated for C_26_H_32_N_2_O_6_SNa^+^ ([M + Na]^+^) 523.19; found: 523.92. The Fmoc,Boc-diprotected l-α-methyl-thialysine: ESI-MS calculated for C_26_H_32_N_2_O_6_SNa^+^ ([M + Na]^+^) 523.19; found: 523.69. The Fmoc,Boc-diprotected l-thialysine: ESI-MS calculated for C_25_H_30_N_2_O_6_SNa^+^ ([M + Na]^+^) 509.17; found: 509.20. (c) To the preliminarily purified intermediate from the last step was added a 50% (*v*/*v*) solution of TFA in DCM (4 mL), and the resulting solution was then stirred at room temperature for 3 h before being concentrated under reduced pressure. (d) To the obtained residue was added a 5% (*w*/*v*) aqueous solution of Na_2_CO_3_ (3 mL) and 1,4-dioxane (3 mL), and to the stirred mixture was added Alloc-OSu (1.5 mmol) at 0 °C in small portions. The reaction mixture was then stirred at room temperature for 5 h, acidified at 0 °C with a 6N hydrochloric acid solution, diluted with the addition of ddH_2_O (20 mL), and extracted with DCM (3 × 10 mL). The combined organics were washed with ddH_2_O and brine, dried over anhydrous Na_2_SO_4_, filtered, and concentrated under reduced pressure. The Fmoc,Alloc-diprotected final products in the respective residues thus obtained were isolated also by semi-preparative silica gel TLC developed with methanol/DCM (1/10, *v*/*v*), and each desired product was eluted out of the excised silica gel band also with methanol/DCM (1/10, *v*/*v*). The three obtained purified samples were then used in the SPPS. The Fmoc,Alloc-diprotected d-α-methyl-thialysine (~28%, overall yield): ^1^H-NMR (400 MHz, CD_3_OD) δ (ppm) 7.71 (d, *J* = 8.0 Hz, 2H, H_arom_), 7.60 (d, *J* = 8.0 Hz, 2H, H_arom_), 7.31 (t, *J* = 8.0 Hz, 2H, H_arom_), 7.24 (t, *J* = 8.0 Hz, 2H, H_arom_), 5.84–5.78 (m, 1H, H_alkene(internal)_), 5.19 (d, *J* = 16.0 Hz, 1H, H_alkene(terminal)_), 5.07 (d, *J* = 8.0 Hz, 1H, H_alkene(terminal)_), 4.50–4.10 (m, 5H, fluorenyl-H_9_, CH_2_O (on Fmoc), and CH_2_O (on Alloc)), 3.35–3.00 (m, 4H, CH_2_NH and S-CH_2_-C_α_), 2.55 (bs, 2H, S-CH_2_), 1.48 (s, 3H, CH_3_); ^13^C-NMR (101 MHz, CD_3_OD) δ (ppm) 176.5 (COOH), 158.7 (NHC(=O)O), 157.2 (NHC(=O)O), 145.4 (C_arom_), 142.7 (C_arom_), 134.5 (C_arom_), 128.9 (C_arom_), 128.3 (C_arom_), 126.4 (C_arom_), 121.0 (C_alkene_), 117.6 (C_alkene_), 67.9 (CH_2_O), 66.5 (CH_2_O), 48.5 (fluorenyl-C_9_), 41.8 (CH_2_NH), 39.2 (CH_2_S), 34.1 (CH_2_S), 23.7 (CH_3_); HRMS (ESI) calculated for C_25_H_29_N_2_O_6_S^+^ ([M + H]^+^) 485.1741; found: 485.1737. The Fmoc,Alloc-diprotected l-α-methyl-thialysine (~31%, overall yield): ^1^H-NMR (400 MHz, CD_3_OD) δ (ppm) 7.64 (d, *J* = 8.0 Hz, 2H, H_arom_), 7.53 (d, *J* = 4.0 Hz, 2H, H_arom_), 7.24 (t, *J* = 8.0 Hz, 2H, H_arom_), 7.17 (t, *J* = 8.0 Hz, 2H, H_arom_), 5.77–5.70 (m, 1H, H_alkene(internal)_), 5.11 (d, *J* = 16.0 Hz, 1H, H_alkene(terminal)_), 5.00 (d, *J* = 12.0 Hz, 1H, H_alkene(terminal)_), 4.45–4.05 (m, 5H, fluorenyl-H_9_, CH_2_O (on Fmoc), and CH_2_O (on Alloc)), 3.25–2.90 (m, 4H, CH_2_NH and S-CH_2_-C_α_), 2.47 (bs, 2H, S-CH_2_), 1.40 (s, 3H, CH_3_); ^13^C-NMR (101 MHz, CD_3_OD) δ (ppm) 158.7 (NHC(=O)O), 157.2 (NHC(=O)O), 145.4 (C_arom_), 142.7 (C_arom_), 134.5 (C_arom_), 128.9 (C_arom_), 128.3 (C_arom_), 126.4 (C_arom_), 121.0 (C_alkene_), 117.6 (C_alkene_), 67.9 (CH_2_O), 66.5 (CH_2_O), 48.5 (fluorenyl-C_9_), 41.8 (CH_2_NH), 39.2 (CH_2_S), 34.1 (CH_2_S), 23.7 (CH_3_); HRMS (ESI) calculated for C_25_H_29_N_2_O_6_S^+^ ([M + H]^+^) 485.1741; found: 485.1730. The Fmoc,Alloc-diprotected L-thialysine (~25%, overall yield): ^1^H-NMR (400 MHz, CD_3_OD) δ (ppm) 7.67 (d, *J* = 8.0 Hz, 2H, H_arom_), 7.57 (dd, *J* = 8.0, 4.0 Hz, 2H, H_arom_), 7.27 (t, *J* = 8.0 Hz, 2H, H_arom_), 7.20 (t, *J* = 8.0 Hz, 2H, H_arom_), 5.84–5.75 (m, 1H, H_alkene(internal)_), 5.17 (d, *J* = 16.0 Hz, 1H, H_alkene(terminal)_), 5.05 (d, *J* = 8.0 Hz, 1H, H_alkene(terminal)_), 4.50-4.05 (m, 6H, fluorenyl-H_9_, CH_2_O (on Fmoc), CH_2_O (on Alloc), and H_α_), 3.20 (t, *J* = 8.0 Hz, 2H, CH_2_NH), 2.98 (dd, *J* = 4.0, 4.0 Hz, 1H, S-CH_2_-C_α_), 2.81 (dd, *J* = 8.0, 8.0 Hz, 1H, S-CH_2_-C_α_), 2.58 (t, *J* = 8.0 Hz, 2H, S-CH_2_); ^13^C-NMR (101 MHz, CD_3_OD) δ (ppm) 174.1 (COOH), 158.8 (NHC(=O)O), 158.6 (NHC(=O)O), 145.3 (C_arom_), 142.7 (C_arom_), 134.5 (C_arom_), 128.9 (C_arom_), 128.3 (C_arom_), 126.4 (C_arom_), 121.0 (C_alkene_), 117.7 (C_alkene_), 68.3 (CH_2_O), 66.5 (CH_2_O), 55.6 (C_α_), 48.4 (fluorenyl-C_9_), 41.6 (CH_2_NH), 34.8 (CH_2_S), 33.3 (CH_2_S); HRMS (ESI) calculated for C_24_H_27_N_2_O_6_S^+^ ([M + H]^+^) 471.1584; found: 471.1580. It should be noted that the ^13^C-NMR signals for C_α_ and COOH (directly linked to C_α_) in the Fmoc,Alloc-diprotected d-α-methyl-thialysine and the Fmoc,Alloc-diprotected l-α-methyl-thialysine were of low intensity or were absent altogether.

#### 3.2.2. Preparation of the Stapled Peptides **2**–**6**

A linear peptide chain bearing all the side chain protecting groups, an acetylated *N*-terminus, and d-α-methyl-thialysine(Alloc) (or l-α-methyl-thialysine(Alloc) or l-thialysine(Alloc)) at the *i* and *i*+4 positions was initially built on the Rink amide MBHA resin employing the Fmoc chemistry-based manual SPPS. For amino acid couplings, 4 equivalents of a *N*^α^-Fmoc-protected amino acid (or ~1 equivalent of the Fmoc,Alloc-diprotected d-/l-α-methyl-thialysines), 3.8 equivalents of the coupling reagent HBTU (or 1 equivalent of the coupling reagent HATU for the double coupling of the Fmoc,Alloc-diprotected d-/l-α-methyl-thialysines) and 3.8 equivalents of the additive HOBt (used only for the HBTU-mediated amino acid couplings) were used in the presence of 0.4 N NMM/DMF. An amino acid coupling reaction was allowed to proceed at room temperature for 1 h (or 9 h for the coupling of the Fmoc,Alloc-diprotected d-/l-α-methyl-thialysines). A 2% (*v*/*v*) DBU/DMF solution was used for Fmoc removal (2 × 5 min at room temperature) [21]. It should be noted that HATU was used as the coupling reagent for the double coupling of the amino acid immediately *N*-terminal to d-α-methyl-thialysine(Alloc) (or l-α-methyl-thialysine(Alloc)), i.e., K and W. The two side chain Alloc groups at the *i* and *i*+4 positions were orthogonally removed in the presence of all the other side chain protecting groups by the treatment with phenylsilane (PhSiH_3_) (24 equivalents) in DCM (2 min at room temperature) followed by the co-treatment with tetrakis(triphenylphosphine)palladium (Pd(PPh_3_)_4_) (0.25 equivalents) in DCM (30 min at room temperature) [18]. The resulting resin with two exposed free amino groups was subsequently treated with *para*-phenylenediacetic acid (1 equivalent) at room temperature for 1 h in the presence of HBTU (2 equivalents) and 0.4 M NMM/DMF, giving rise to the resin-bound side chain fully protected stapled peptide. Following treating such a peptidyl resin with Reagent K (83.6% (*v*/*v*) TFA, 5.9% (*v*/*v*) phenol, 4.2% (*v*/*v*) ddH_2_O, 4.2% (*v*/*v*) thioanisole, 2.1% (*v*/*v*) ethanedithiol) at room temperature for 4 h, the reaction mixture was filtered and the volatiles in the filtrate were evaporated away with a stream of nitrogen gas in a well-ventilated fume hood. To the resulting residue was added cold diethyl ether to precipitate out the crude peptides **2**–**6** which were then all purified by semi-preparative RP-HPLC on a C18 column (1 × 25 cm, 5 µm) eluted at 4.5 mL/min with ultraviolet monitoring at 214 nm with the following gradient of ddH_2_O containing 0.05% (*v*/*v*) of TFA (mobile phase A) and acetonitrile containing 0.05% (*v*/*v*) of TFA (mobile phase B): linear increase of B from 0% to 35% in 60 min. The pooled desired HPLC fractions were concentrated under reduced pressure to remove volatiles and the remaining aqueous solution was lyophilized to afford the purified stapled peptides **2**–**6** all as puffy white solids. Of note, the purified stapled peptides **2**–**6** were obtained in overall yields of ~1.2–2.4%; while yield improvement was not attempted in the current study, it would be a worthwhile future endeavor. The purified stapled peptides **2**–**6** were all >95% pure based on RP-HPLC analysis on an analytical C18 column (0.46 × 25 cm, 5 μm) eluted at 1.0 mL/min with ultraviolet monitoring at 214 nm with the following gradient of the afore-mentioned mobile phases A and B: linear increase of B from 0% to 40% in 60 min; under such conditions, **2**–**6** respectively appeared at the following retention times: 47.2 min, 47.8 min, 47.1 min, 49.5 min, and 48.0 min. The exact masses of the purified stapled peptides **2**–**6** were all confirmed by electrospray ionization–MS analysis (Table 1).

#### 3.2.3. Preparation of the Stapled Peptides **7** and **8**

These two peptides were prepared in the same manner as that for the stapled peptides **2**–**6** described above and in Scheme 2. The pre-requisite Fmoc,Alloc-diprotected l-α-methyl-thialysine and the Fmoc,Alloc-diprotected l-thialysine were prepared according to Scheme 1. The crude peptides **7** and **8** were purified by semi-preparative RP–HPLC with the largely same conditions as that described above for the purification of the stapled peptides **2**–**6**, except for using the following gradient of the mobile phases A and B: linear increase of B from 0% to 60% in 60 min. The exact masses of the purified stapled peptides **7** and **8** were also confirmed by MS analysis (Table 1). The purified **7** and **8** were each also >95% pure based on a RP–HPLC analysis with the largely same condition as that described above for the RP–HPLC analysis of the purified stapled peptides **2**–**6**, except for using the following gradient of the mobile phases A and B: linear increase of B from 0% to 60% in 60 min**;** under such conditions, **7** and **8** respectively appeared at the following retention times: 46.9 min and 44.1 min. Of note, the purified **7** and **8** were obtained in overall yields of ~1.5% and ~0.12%, respectively. Again, while yield improvement was not attempted in the current study, it would be a worthwhile future endeavor.

### 3.3. CD Experiment

This experiment for peptides **2**–**8** followed the protocol previously described by our laboratory [22]. Each peptide was assayed in duplicate at 25 μM and/or 50 μM in ddH_2_O. The representative CD spectra for peptides **2**–**6** each at 25 μM are shown in Figure 2 and those for peptides **7** and **8** each also at 25 μM are shown in Figure 4, together with the calculated % α-helicity values. The CD spectra were recorded on a JASCO circular dichroism spectrometer (Model J-815) equipped with a 1-mm path length sample cell. The CD experiment was performed at 25 °C with baseline signal correction from the sample solvent ddH_2_O. The scanning speed, bandwidth, and wavelength range were set at 50 nm/min, 1 nm, and 190–250 nm, respectively. The two sets of mean residue ellipticity [θ] values for each peptide were calculated from the millidegree values from the instrument readout, the molar concentration of the peptide sample, and the number of amino acid residues of the peptide according to the literature equation [19]. The two % α-helicity values for each peptide were calculated according to literature equation [19] from the two mean residue ellipticity [θ] values at 222 nm ([θ]_222_) and the maximum [θ]_222_, and expressed as mean ± standard deviation.

### 3.4. Proteolysis Experiment

This experiment followed the protocol described previously [23]. Fifty (50) µL of an 160 μM solution of a peptide (**5**, **6**, **7**, or **8**) in ddH_2_O was thoroughly mixed with 50 μL of an 8 ng/μL solution of pronase in 100 mM Tris•HCl (pH 7.3), and the resultant solution was incubated at 37 °C until quenched at different time points (0 min, 4 min, 7.5 min, and 15 min) with an 1.0 M solution of acetic acid in ddH_2_O. Twenty (20) μL of a pronase digestion solution was taken at each time point and treated immediately with 40 μL of the 1.0 M acetic acid aqueous solution, followed instantly by vigorous vortexing and centrifugation. The obtained supernatant was then injected into a C18 analytical HPLC column (0.46 × 25 cm, 5 μm), and the column was eluted at 1 mL/min with ultraviolet monitoring at 214 nm with the following gradients of ddH_2_O containing 0.05% (*v*/*v*) TFA (mobile phase A) and acetonitrile containing 0.05% (*v*/*v*) TFA (mobile phase B): linear increase of B from 0% to 45% in 60 min (for **5** and **6**), linear increase of B from 0% to 60% in 60 min (for **7**), or linear increase of B from 0% to 100% in 60 min (for **8**). The integrated HPLC peak areas at different time points for a given peptide were used to estimate the percentage remaining for the peptide as the function of the digestion time.

## 4. Conclusions

To summarize, in the current study, by employing the up-to-date most efficacious bis-lactam [*i*, *i*+4]-stapling system discovered recently in our laboratory, which was constructed on a model peptide sequence corresponding to amino acids 541–558 of p110α[E545K], we found that the bis-lactam [*i*, *i*+4]-stapled peptides with d- or l-α-methyl-thialysines (i.e., **2**–**5**) exhibited largely comparable % α-helicity to each other, yet greater % α-helicity than the bis-lactam [*i*, *i*+4]-stapled peptide with L-thialysine (i.e., **6**). An l-α-methyl-thialysine-stapled peptide built on a model peptide sequence derived from RNase A was also found to exhibit a greater % α-helicity than its l-thialysine-stapled counterpart. Moreover, the l-α-methyl-thialysine-stapled p110α[E545K] and RNase A peptides were both found to be proteolytically more stable than their respective l-thialysine-stapled counterparts. Therefore, our findings in the current study may be applicable to other peptide sequences as well.

## Figures and Tables

**Table 1:** MS analysis of peptides **2**–**8** ^a^

Peptide	Ionic Species	Calculated *m*/*z*	Observed *m*/*z*
**2**	[M + 2H]^2+^ [M + 3H]^3+^ [M + 4H]^4+^	1312.62 875.42 656.82	1313.63 876.89 657.98
**3**	[M + 2H]^2+^ [M + 3H]^3+^ [M + 4H]^4+^	1312.62 875.42 656.82	1313.33 876.41 657.64
**4**	[M + 2H]^2+^ [M + 3H]^3+^ [M + 4H]^4+^ [M + 5H]^5+^	1312.62 875.42 656.82 525.65	1313.72 876.81 658.02 526.70
**5**	[M + 2H]^2+^ [M + 3H]^3+^ [M + 4H]^4+^ [M + 5H]^5+^	1312.62 875.42 656.82 525.65	1313.68 876.35 657.95 526.70
**6**	[M + 2H]^2+^ [M + 3H]^3+^	1298.61 866.07	1299.67 867.74
**7**	[M + H]^+^ [M + 2H]^2+^	1963.9 982.5	1964.7 983.1
**8**	[M + H]^+^ [M + 2H]^2+^	1935.9 968.4	1936.5 969.3

^a^ All peptides were measured with electrospray ionization–MS (positive ion mode).
